# Measuring the invisible: perinatal health outcomes of unregistered women giving birth in Belgium, a population-based study

**DOI:** 10.1186/s12884-021-04183-9

**Published:** 2021-10-29

**Authors:** Claudia Schoenborn, Myriam De Spiegelaere, Judith Racape

**Affiliations:** 1 Research centre in Social Approaches to Health, School of Public Health, Université libre de Bruxelles (ULB), Bruxelles, Belgium; 2 Research centre in Epidemiology, Biostatistics and Clinical Research, School of Public Health, Université libre de Bruxelles (ULB), Bruxelles, Belgium; 3 Chair in Health and Precarity. Faculty of Medecine, Université libre de Bruxelles (ULB), Bruxelles, Belgium

**Keywords:** Perinatal health, Health inequalities, Undocumented migrants, Socioeconomic status, Vital statistics, Unregistered population

## Abstract

**Background:**

The unregistered population remains under-researched because of its “invisible” status in statistics. Studies on perinatal health outcomes of unregistered women remains particularly limited. Our objectives were 1) to describe the sociodemographic profiles of women who are not legally residing in Belgium and 2) to analyze the associations of registration status with pregnancy outcomes according to socioeconomic status and nationality.

**Methods:**

We analysed data from birth and death certificates taken from the Belgian civil registration system, linked with the National Population Registry (NPR). The data relates to all singleton babies born between 2010 and 2016 (*n* = 871,283), independent of their mother’s NPR registration status. We used logistic regression to estimate the odds ratios for the associations between perinatal outcomes (perinatal mortality, prematurity and low birth weight) and maternal NPR registration status according to socioeconomic status and maternal nationality.

**Results:**

Over the study period, 1.9% of births were to mothers without NPR-registration. Unregistered women from newer EU member states and non-European countries were particularly disadvantaged from a socioeconomic point of view. Apart from women with a South American nationality, all other groups of unregistered women had higher rates of prematurity, low birth weight, and perinatal mortality, compared to registered mothers (*p* < 0.0001). Unregistered women from Belgium and EU15 nationalities had particularly higher rates of prematurity, low birth weight, and perinatal mortality, compared to registered mothers, even after adjustment for socioeconomic status (*p* < 0.0001). The excess of perinatal mortality for non-European unregistered mothers could partly be explained by their precarious socioeconomic situation.

**Conclusions:**

This is the first study to include data on mothers who were not legally residing in Belgium. Unregistered women giving birth in Belgium are likely a heterogeneous socioeconomic group. Overall, unregistered women have increased risks of adverse perinatal outcomes, but it is likely that the causal mechanisms differ starkly between Belgian, European and non-European women. Further research is needed to understand the mechanisms behind these accrued rates. It is important to keep measuring the health outcomes of the populations which are “invisible” in national statistics, in order to identify the groups in most need of integration and access to services.

**Supplementary Information:**

The online version contains supplementary material available at 10.1186/s12884-021-04183-9.

## Introduction

The population without legal status remains under-researched because it is not included in population databases and statistics due to their “invisible” status. Data of unregistered populations are often based on estimates or surveys. The main function of civil registration is to provide individuals with documentation needed to establish legal identity, make claims of nationality, access health services, exercise civil and political rights [1].

Like in other countries, every year in Belgium, the national statistical office publishes the data for perinatal health outcomes of births within its territory [2]. Previous epidemiologic studies have analysed these data, which revealed social inequalities for various perinatal health indicators both related to immigration and, especially, to the socioeconomic status of mothers [3–7]. This routinely collected data however only concerns legal residents in Belgium. All women not officially registered in the National Population Registry (NPR), such as undocumented migrants (UMs), women without stable accommodation (homeless, traveller populations), and women residing abroad but giving birth in Belgium are thus excluded from these statistics. The former two groups, undocumented migrants and women without stable accommodation, are likely to be particularly vulnerable from a socioeconomic and perinatal health point of view.

Pregnant undocumented migrants appear to be exposed to a triple burden: first, they are a group of particularly vulnerable migrants [8] who have likely suffered traumatic experiences before or during migration [9]. Second, without a legal status, their civil, political, and economic rights in their new home country are severely limited; and crucially for their health and that of their unborn children, their access to perinatal care is complicated at best [10, 11]. Third, being undocumented exposes women to poor living and working conditions, to isolation, discrimination, instability, and anxiety [12]. All these effects of living without a legal status can have profound implications for their physical and mental health in general [13], and for their perinatal health more specifically. Overall, the literature shows that undocumented women are at higher risk of severe maternal morbidity and mortality, and of having premature or low birthweight babies [14, 15]. Because of their invisibility in administrative data, the exact number of people without a residency permit is unknown. Studies estimate that around 1% of the population in the European Union and almost 20% of the non-EU-EFTA immigrants are undocumented [16]. In Belgium, it has been estimated that undocumented migrants represent more than 1% of the entire population [9].

The research on the perinatal health outcomes of unregistered women remains limited [14]. This study aims to analyze perinatal health outcomes in an exhaustive Belgian population-based database which includes mothers who were not legally residing in Belgium. The objectives of our work are to describe the socioeconomic profiles (overall, and by nationality group) of women who are not registered in Belgium, and to quantify the associations of the unregistered status with pregnancy outcomes (perinatal mortality, prematurity and low birth weight).

## Methods

### Study population and data

We used data from birth and death certificates taken from the Belgian civil registration system which had been linked with the National Population Registry to obtain the maternal registration status. The data relates to all singleton babies born between 1st January 2010 and 31st December 2016, independent of their mothers’ NPR registration status (thus including for example asylum seekers who have temporary national registration numbers, and undocumented residents who have none).

In Belgium, the medical data concerning all live births and deaths from 22 completed weeks of gestation until 1 year of age, or a birth weight > 500 g are registered in hospital by midwives and obstetricians. Socio-economic data, including nationality, are reported by the parents at the civil registration service within 15 days of the birth. In Belgium, two perinatal epidemiological centers, SPE (Studiecentrum voor Perinatale Epidemiologie) for the Dutch-speaking region and CEpiP (Centre d’Epidémiologie Périnatale) for the French-speaking region, are responsible for the quality and the completeness of the data encoded in birth and death certificates.

All people legally residing in Belgium and asylum-seekers awaiting a decision are registered in the NPR with a National Registration Number. Belgian nationals living abroad and registered at foreign consulates are also, in principle, included in the national registry, although they can sometimes absent from the registry. Registering in the NPR is conditional to a person’s right of residence in Belgium and to a proof of residence (including an investigation carried out by the municipal authorities to verify the residence). Registration in the NPR gives access to a series of rights and social security services, including public health insurance. Healthcare services in Belgium are mostly publicly funded and are theoretically accessible to anyone, but reimbursement of costs is only possible via public or private health insurance.

In this article, we will refer to women legally residing in Belgium, thus registered in the National Population Registry, as “NPR registered”, and those who do not figure in the NPR as “NPR unregistered”.

The data collection was carried out by Statbel (the Belgian statistical office) and was exempted by law from requiring ethical approval. The process of obtaining data from death and birth certificates of the Belgian civil registration system is regulated by the Belgian Commission for the Protection of Privacy, which approved this study. Statistics Belgium provided two databases. Statbel routinely links the medical data from birth and death certificates with the socioeconomic data from the birth/death declarations made by parents at the civil registration service, via the maternal national registration number. Statbel provided us with two anonymised databases for the period 01/01/2010 to 31/12/2016: 1) all live births (*n* = 898,616), and 2) all deaths of babies before 1 year of age (*n* = 8075). The maternal national registration numbers were encrypted, i.e. replaced with a unique identifier for each birth). For each birth/death, one variable specified whether or not the mother had a national registration number, which allowed us to make the distinction between NPR registered and non-NPR registered mothers. We were not able to identify births to the same mother within the seven-year period, given that the unique identifier related to births and not to mothers. We excluded multiple births.

For mothers with NPR registration, we performed the linkage between the live births and deaths via the “merge” function in STATA v16 by using the unique identification number. Given that for stillbirths there was only a death certificate, no linkage was needed in these cases .

For the 16,594 births without NPR registration, there were 16,119 live births, 193 stillbirths, so only 282 needed linking between birth and death certificates. We linked live births and neonatal/infant deaths for mothers without a NPR number (*n* = 282) based on the baby’s date of birth, birth weight, gestational age, and parity. *N* = 67 births could not be linked via these variables alone and were thus linked manually, taking into account additional variables such as sex of the baby, maternal age, and obstetric interventions. We were able to link every single record.

### Definitions of the exposure and outcome variables

From the birth certificates we extracted the following data concerning the baby: gestational age at birth, birth weight, Apgar score, and congenital anomalies. The following data was extracted concerning the mother: age, parity, diabetes, hypertension, mode of delivery, nationality, educational level, her and her partner’s employment status, and if living with a partner or not.

We computed perinatal mortality, defined as deaths from 22 weeks of gestation until 7 days after birth. We defined low birth weight (LBW) as a weight below 2500 g and prematurity when gestational age was inferior to 37 completed weeks. Results for LBW follow a comparable pattern to those of prematurity.

Parity was categorised into three groups: nulliparity, 1 or 2 previous births, and 3 or more previous births. Age of the mother was also categorised into three groups: < 20 years, 20 to 39 years, and ≥ 40 years.

Maternal nationality refers to the nationality she had at her child’s birth. We classified nationalities into 11 groups, namely Belgium, members of the European Union EU15, EU27 without EU15 countries, Eastern European countries, Turkey, Maghreb, sub-Saharan Africa, North Asia and Middle east, South America, other countries, and unknown (Supplementary data 1). In Belgium, nationality is linked to immigration status, working rights, legal and social rights, more than it is to ethnicity.

The maternal level of education was categorised into four groups: tertiary (university or higher education), secondary (completed secondary school), primary (completed primary or less), and unknown.

Data on paternal and maternal employment status (employed or not) were combined to derive the number of household incomes that stemmed from employment (two, one, or none). For mothers living without a partner, the paternal status was not taken into account, so in this case the number of parents employed could only vary between zero and one.

### Statistical analysis

Categorical variables are presented with percentages and compared between registered and unregistered mothers by using Pearson Chi^2^ test. We used logistic regression to estimate the odds ratios (ORs) for the associations between NPR registration status and perinatal outcomes (LBW, prematurity and perinatal mortality), stratified by nationality. First, the crude associations between NPR registration status and perinatal outcomes were estimated. Then, multivariate models were developed to assess the effects of potential confounding variables: parity, maternal age and two SES variables (employment status and maternal education). The Hosmer and Lemeshow test was used to check the goodness-of-fit of the model. We present the odds ratios and their 95% confidence intervals derived from the logistic regressions and the *p*-values for the Wald χ^2^ test. The significance level was set at α = 0.05, and all analyses were performed using Stata 16 software.

## Results

### Maternal socioeconomic characteristics and perinatal outcomes according to NPR registration status (Table 1)

Over the period 2010–2016, a total of 1.9% of singleton births (*n* = 16,594) were to mothers who were not registered in the NPR. A higher proportion of unregistered mothers lived without a partner (33.7% vs 12.3% of registered mothers) and had a primary educational level (11.4% vs 4.7%). Nearly 7% of unregistered mothers were under 20 years of age (vs. 2.8% for registered mothers); they were less likely to have two household incomes and more likely to have no incomes at all (38.6% vs. 61.7, and 36% vs. 13% respectively). The most represented nationalities among unregistered women were EU15 (25.5%), Belgium (18.7%), and EU27 (14%).

**Table 1:** Maternal sociodemographic characteristics and perinatal outcomes according to the NPR registration status

n(%)	NPR registration ***N*** = 854,689	No NPR registration ***N*** = 16,594	Relative prevalence (% in NPR-unregistered / %in NPR-registered)
**% of births**	98.1	1.9	
**Parity**			
Nulliparity	371,179 (43.8)	7513 (46)	1.05
1–2	404,531 (47.7)	7346 (45)	0.95
≥ 3	71,988 (8.5)	1464 (9.0)	1.06
*missing*	0,8%	1.6%	
**Living alone**			
Yes	103,633 (12.3) 1.4%	5311 (33.7)	2.74
*missing*		4.9%	
**Maternal age (years)**			
≥ 40	15,324 (1.8)	429 (2.6)	1.44
20 < 40	815,185 (95.45)	14,990 (90.45)	0.95
< 20	23,497 (2.75)	1151 (6.95)	2.53
*missing*	0.08%	0.1%	
**Maternal education**			
≤ primary	40,517 (4.7)	1893 (11.4)	2.41
secondary)	369,011 (43.2)	6448 (38.9)	0.90
tertiary	339,370 (39.7)	4307 (26)	0.65
Other/ Unknown	105,791 (12.4)	3946 (23.8)	1.92
**Number of incomes**			
0 incomes	104,200 (13.2)	4822 (36.4)	2.76
1 income	197,341 (25.1)	3303 (25.0)	1.00
2 incomes	485,717 (61.7)	5108 (38.6)	0.63
*missing*	7.9%	20.2%	
**Nationality**			
Belgium	663,539 (77.6)	3107 (18.7)	0,24
EU15	54,895 (6.4)	4239 (25.5)	3,98
EU27 without EU15	27,724 (3.2)	2324 (14.0)	4,38
Eastern Europe	13,754 (1.6)	1430 (8.6)	5,38
Turkey	8212 (0.96)	228 (1.4)	1,46
Maghreb	33,570 (3.9)	1316 (7.9)	2,03
Sub-Saharan Africa	24,932 (2.9)	1445 (8.7)	3,00
South America	4200 (0.5)	816 (4.9)	9.80
Middle east and North/West Asia	10,546 (1.2)	584 (3.5)	2.92
Other	10,580 (1.2)	623 (3.75)	3.13
Unknown	2737 (0.3)	482 (2.9)	9.67
**Congenital anomalies**			
Yes	5892 (0.7)	316 (1.9)	2.76
*missing*	1.8%	1.6%	
**Hypertension**			
Yes	37,638 (4.5)	656 (4.1)	0.91
*missing*	2.1%	3.7%	
**Diabetes**			
Yes	43,960 (5.3)	958 (6.1)	1.15
*missing*	2.5%	5.6%	
**Mode of delivery**			
Vaginal	672,570 (80.2)	12,664 (77.7)	0.97
Planned caesarean	84,184 (10.0)	1699 (10.4)	1.04
Emergency caesarean	81,834 (9.8)	1928 (11.8)	1.20
*missing*	1.9%	1.8%	
**Perinatal mortality**			
Yes	5311 (0.62)	334 (2.01)	3.24
**Stillbirth**			
Yes	4170 (0.49)	193 (1.16)	2.37
**Birth weight**			
< 2500 g	46,061 (5.5)	1491 (9.0)	1.65
2500 < 4000 g	727,578 (86.4)	13,836 (83.8)	0.97
≥ 4000 g	68,757 (8.2)	1188 (7.2)	0.88
*missing*	1.4%	0.5%	
**Prematurity**			
< 37	55,771 (6.6)	1742 (10.6)	1.61
*missing*	1.4%	0.6%	
**Apgar 5 min**			
< 7	16,576 (1.97)	744 (4.5)	2.28
*missing*	1.5%	0.9%	

*p < 0.0001 for all, except hypertension (p = 0.02)*

Mothers with unregistered status had significantly higher rates of adverse perinatal outcomes (*p* < 0.0001): LBW (9% vs 5.5%), prematurity (10.6% vs 6.6%), perinatal mortality (2% vs 0.6%), stillbirth (1.2 vs 0.5%), Apgar score < 7 (4.5% vs 2%), emergency caesarean (11.8% vs 9.8%), and congenital anomalies (1.9% vs 0.7%).

### Unregistered mothers’ socioeconomic characteristics and perinatal outcomes by nationality group (Table 2)

The nationality groups with the highest proportions of unregistered mothers were South America (16%), unknown (15%), and Eastern Europe (9.4%). There were very high proportions of mothers living without a partner among those with a nationality from Sub-Saharan Africa (59%) and from EU27 (excluding EU15) member states (51.6%). Nearly 21% of the latter group were also younger than 20 years old. Mothers from Eastern Europe, the Middle East, Sub-Saharan Africa, Turkey, and Maghreb were particularly at risk of having a very low educational level (19–22%). Inequalities in terms of income were stark: in contrast to mothers with a Belgian or EU15 nationality, huge proportions of unregistered migrant mothers had no household income (71% of mothers from Eastern Europe, and around 65% of mothers from Maghreb and Sub-Saharan Africa).

**Table 2:** Distribution of socioeconomic and clinical characteristics of unregistered mothers, by nationality group and Relative Prevalence (RP) compared with NPR registered mother

N = 16,594	Belgium (***n*** = 3107)		EU15 (***n*** = 4239)		EU27 without EU15 (***n*** = 2324)		East Europe (***n*** = 1430)		Turkey (***n*** = 228)		Maghreb (***n*** = 1316)		Sub-Saharan Africa (***n*** = 1445)		South America (***n*** = 816)		Middle east (***n*** = 584)		Other (***n*** = 623)		Unknown (***n*** = 482)	
% of births	0.47	RP	7.2	RP	7.7	RP	9.4	RP	2.7	RP	3.8	RP	5.5	RP	16.3	RP	5.2	RP	5.6	RP	15.0	RP
**Parity**	3079		4094		2313		1421		225		1296		1435		811		573		621		455	
Nulliparity (%)	45.6	1..03	45.9	1..02	49.3	1..03	44.1	1.17	38.7	1.12	49.9	1.31	42.2	1.26	49.8	1.09	37.7	1.02	44.9	0.88	49.5	1.47
≥3 children (%)	8.1	1..02	7.6	0..90	8.3	1..42	12.0	0.72	12.9	1.45	9.2	0.77	12.3	0.79	6.0	0.87	12.4	0.88	5.8	1.35	13.6	0.61
**Living alone**	3066		4120		2223		1358		222		1262		1380		795		538		590		223	
Yes (%)	17.3	1..47	22.8	2..15	51.6	4..06	43.7	2.57	35.1	6.50	38.3	6.60	59.5	1.58	38.5	2.79	25.7	2.07	29.3	4.37	45.3	1.32
**Maternal age**	3107		4238		2324		1426		228		1310		1444		816		583		623		471	
< 20 (%)	2.5	0..96	3.1	1..82	20.7	4..22	14.9	1.57	5.7	1.43	2.4	1.50	4.1	1.12	6.9	2.30	3.4	0.83	1.4	2.09	12.5	1.38
≥ 40 (%)	2.1	1..31	3.9	1..11	1.4	0..88	1.3	0.93	2.2	1.57	4.05	1.35	2.2	1.05	1.7	0.50	1.7	1.06	3.5	1.35	2.3	0.82
**Maternal education**	3107		4239		2324		1430		228		1316		1445		816		584		623		482	
< primary (%)	1.7	0..74	2.5	0..78	18.3	1..91	21.9	1.06	21.9	1.03	22.1	0.98	20.9	1.05	19.0	2.60	15.1	0.71	7.5	0.82	12.7	0.92
secondary (%)	42.9	0..99	45.6	1..19	29.2	0..63	34.4	0.88	32.5	0.64	40.0	0.84	36.1	0.81	54.7	1.11	27.9	0.79	28.1	0.84	21.8	1.11
tertiary (%)	41.9	0..95	35.3	0..89	20.4	0..75	11.1	0.74	18.9	3.00	10.3	0.95	14.6	1.11	11.1	0.41	24.5	1.54	35.4	0.99	7.1	2.63
Other (%)	13.5	1..36	16.7	0..89	32.1	1..91	32.6	1.29	26.8	1.25	27.7	1.44	28.4	1.26	15.2	0.94	32.5	1.17	28.9	1.33	58.5	0.92
**Number of incomes**	2817		3735		1806		1001		172		945		1118		625		427		467		120	
0 incomes (%)	10.3	0..98	14.3	1..32	57.6	4..40	71.4	1.66	52.3	2.14	63.7	2.46	64.9	1.39	58.9	3.73	52.7	1.25	34.9	2.92	57.5	0.86
1 income (%)	23.0	1..14	27.5	0..91	21.9	0..56	19.9	0.47	33.1	0.52	28.3	0.44	22.5	0.55	26.7	0.59	26.7	0.57	33.0	0.60	20.0	0.71
2 incomes (%)	66.7	0..96	58.3	0..99	20.4	0..43	8.7	0.59	14.5	1.26	12.5	1.19	12.5	0.99	20.6	0.53	20.6	1.91	32.1	0.97	22.5	4.50
**Congenital anomalies**	3082		4096		2314		1421		226		1297		1436		812		575		621		455	
Yes (%)	2.3	3..33	2.6	4..00	1.4	2..19	1.1	1.47	1.9	1.90	1.6	1.82	0.97	1.35	0.99	1.46	2.4	3.24	1.3	2.10	5.5	5.24
**Hypertension**	3039		4045		2179		1377		220		1281		1417		802		565		618		437	
Yes (%)	4.5	0..94	3.9	1..08	2.5	0..74	3.5	1.35	1.4	0.52	4.3	1.95	7.1	1.03	6.5	1.67	1.1	0.50	4.05	1.62	3.9	1.03
**Diabetes**	2977		4012		2092		1347		215		1247		1376		797		554		613		428	
Yes (%)	5.0	1..03	4.7	0..87	4.0	0..78	4.6	1.07	4.2	0.70	14.3	1.34	7.3	0.94	8.7	1.28	7.0	1.04	7.5	1.07	8.4	0.97
**Delivery Mode**	3065		4087		2310		1419		225		1296		1434		811		573		620		451	
Vaginal (%)	78.9	0..98	77.1	0..97	84.8	1..05	83.4	0.99	78.7	0.96	74.5	0.91	67.5	0.98	72.8	1.03	76.6	0.96	72.9	0.98	80.9	1.03
Caesarean (%)	10.1	1..01	12.2	1..17	6.5	0..68	6.8	0.88	12.9	1.45	10.6	1.28	14.2	1.05	11.8	0.77	11.7	1.07	12.6	1.07	8.0	0.74
Emergency caesarean (%)	11.1	1..18	10.8	1..05	8.7	0..92	9.8	1.26	8.4	0.91	14.9	1.48	18.3	1.05	15.4	1.10	11.7	1.22	14.5	1.06	11.1	1.02
**Perinatal mortality**	3107		4239		2324		1430		228		1316		1445		816		584		623		482	
Yes (%)	2.2	4..31	3.6	7..06	0.86	1..62	0.84	1.62	1.3	1.76	1.3	1.71	1.7	1.87	0.74	1.07	1.0	1.30	0.80	1.51	4.4	0.17
**Low birth weight**	3087		4217		2316		1425		226		1310		1438		814		578		622		472	
Yes (%)	9.6	1..75	10.4	2..08	8.8	1..96	7.0	1.63	4.0	0.82	5.65	1.53	10.2	1.55	5.5	1.10	7.4	1.38	7.7	1.45	18.4	0.49
**Prematurity**	3084		4217		2310		1425		225		1310		1440		814		578		622		472	
Yes (%)	10.7	1..57	11.7	2..03	12.2	2..07	7.65	1.39	7.6	1.31	7.5	1.69	11.0	1.55	7.7	1.15	8.65	1.52	7.7	1.28	19.7	0.53

Mothers with the highest rates of hypertension were from Sub-Sahara Africa (7.1%), the Middle East (6.5%), and Belgium (4.8%). The prevalence of diabetes was highest in Maghrebi women (14.3%); congenital anomalies were most prevalent in women from EU15 (2.6%) and Belgium (2.3%); and emergency caesareans were most frequent in Sub-Sahara Africans (18.3%) and south Americans (15.4%).

The relative prevalences of perinatal mortality, prematurity, and low birthweight were higher in unregistered women of all nationality groups, compared to their unregistered counterparts. The exception was low-birth weight which was higher in Turkish NPR-registered women than their unregistered counterparts. Congenital anomalies were also higher in all groups of NPR-unregistered women, compared to those who were registered.

Supplementary Table shows the socioeconomic and perinatal health characteristics of NPR registered women, which enables to draw comparisons for the different nationality groups (Supplementary data 2).

### Perinatal outcomes according to NPR registration status (Tables 3 and 4)

The crude Odd Ratios for perinatal mortality (Table 3) were statistically significantly higher for NPR-unregistered women compared to NPR-registered women with a nationality from Belgium, EU15, EU27, Maghreb, and Sub-Saharan Africa. The nationality groups of unregistered mothers with the strongest ORs were EU15 (OR(95%CI) 7.3 (6.0–8.95), *p* < 0.0001), followed by Belgium (OR(95%CI) 4.3(3.3–5.4), *p* < 0.0001). When adjusting for sociodemographic characteristics, the associations between NPR registration status and perinatal mortality remained significant only for mothers with a Belgian nationality or a nationality from EU15 member states (ORs 3.6 and 3.4 respectively, p < 0.0001).

**Table 3:** Perinatal mortality (crude rates among No maternal NPR) and Odds Ratios according to NPR registration status (reference is maternal NPR), stratified by maternal nationality

Maternal Nationality	Per 1000 births	OR^**a**^	CI (95%)	***p-value***	aOR^**b**^	CI (95%)	***p-value***
**Belgium**		cases = 3476 / *n* = 666,646			cases = 2827 / *n* = 624,389		
	21.6	**4.3**	**3.3–5.4**	***< 0.0001***	**3.6**	**2.7–4.8**	***< 0.0001***
**EU15**		cases = 432 / *n* = 59,134			cases = 251 / *n* = 49,919		
	36.1	**7.3**	**6.0–8.95**	***< 0.0001***	**3.4**	**2.5–4.6**	***< 0.0001***
**EU27 (without EU15)**		cases = 167 / *n* = 30,048			cases = 113 / *n* = 26,426		
	8.6	**1.6**	**1.02–2.6**	***0.04***	1.1	0.6–2.15	*0.74*
**Eastern Europe**		cases = 84 / *n* = 15,184			cases = 59 / *n* = 12,478		
	8.4	1.6	0.95–3.0	*0.13*	1.3	0.5–3.1	*0.60*
**Turkey**		cases = 64 / *n* = 8440			cases = 50 / *n* = 7470		
	13.1	1.8	0.55–5.7	*0.33*	0.79	0.10–6.3	*0.82*
**Maghreb**		cases = 272 / *n* = 34,886			cases = 195 / n = 30,641		
	12.9	**1.7**	**1.04–2.8**	***0.03***	1.6	0.8–3.1	*0.18*
**Sub-Saharan Africa**		cases = 251 / n = 26,377			cases = 151 / n = 22,295		
	16.7	**1.8**	**1.2–2.8**	***0.005***	1.7	0.96–3.1	*0.07*
**South America**		cases = 35 / *n* = 5016			cases = 26 / *n* = 4286		
	7.4	1.1	0.4–2.6	*0.89*	0.91	0.3–2.9	*0.87*
**Middle East and North/West Asia**		cases = 87 / *n* = 11,130			cases = 53 / *n* = 8966		
	10.3	1.3	0.6–3.1	*0.49*	1.4	0.4–4.6	*0.60*
**Other**		cases = 61 / n = 11,203			cases = 46 / *n* = 9654		
	8.0	1.5	0.6–3.8	*0.37*	1.6	0.5–4.6	*0.42*
**Outside EU**		cases = 802 / *n* = 103,028			cases = 541 / n = 87,311		
	11.7	1.6	1.22–1.99	*< 0.0001*	1.5	1.07–2.08	*0.02*

^a^ Crude OR

^b^ Adjusted OR for maternal age, single, number of incomes and maternal education

**Table 4:** Prematurity (crude rates among No maternal NPR) and Odds Ratios) according to registration status (reference is maternal NPR), stratified by maternal nationality

Maternal Nationality	Per 100 births	OR^**a**^	CI (95%)	***p-value***	aOR^**b**^	CI (95%)	***p-value***
**Belgium**		cases = 44,936 / *n* = 661,984			cases = 41,659 / *n* = 626,412		
	10.7	**1.6**	**1.5–1.8**	***< 0.0001***	**1.4**	**1.2–1.6**	***< 0.0001***
**EU15**		cases = 3373 / *n* = 54,330			cases = 2939 / n = 49,798		
	11.7	**2.2**	**1.96–2.4**	***< 0.0001***	**1.7**	**1.5–1.9**	***< 0.0001***
**EU27 (without EU15)**		cases = 1886 / *n* = 29,545			cases = 1611 / n = 26,398		
	12.2	**2.2**	**1.9–2.5**	***< 0.0001***	**1.7**	**1.45–2.05**	***< 0.0001***
**Eastern Europe**		cases = 855 / n = 15,042			cases = 667 / n = 12,470		
	7.65	**1.4**	**1.2–1.8**	***0.001***	1.2	0.95–1.6	*0.11*
**Turkey**		cases = 487 / *n* = 8374			cases = 430/ n = 74,610		
	7.6	1.3	0.8–2.2	*0.26*	0.91	0.44–1.85	*0.79*
**Maghreb**		cases = 1580 / n = 34,647			cases = 1355 / n = 30,611		
	7.5	**1.7**	**1.4–2.1**	***< 0.0001***	**1.6**	**1.25–2.1**	***< 0.0001***
**Sub-Saharan Africa**		cases = 1905 / n = 26,170			cases = 1557 / n = 22,276		
	11.0	**1.6**	**1.4–1.9**	***< 0.0001***	**1.4**	**1.2–1.8**	***< 0.0001***
**South America**		cases = 340 / *n* = 4921			cases = 286 / *n* = 4283		
	7.7	1.15	0.9–1.5	*0.31*	0.98	0.7–1.42	*0.91*
**Middle East and North/West Asia**		cases = 642 / n = 11,008			cases = 489 / *n* = 8953		
	8.65	**1.6**	**1.2–2.1**	***0.003***	**1.6**	**1.1–2.3**	***0.015***
**Other**		cases = 671 / n = 10,934			cases = 567 / *n* = 9643		
	7.7	1.3	0.96–1.8	*0.09*	1.4	1.0–2.01	*0.07*
**Outside EU**		cases = 5914 / *n* = 102,109			cases = 4876 / n = 87,764		
	8.6	1.6	1.44–1.74	*< 0.0001*	1.4	1.23–1.55	*< 0.0001*

^a^ Crude OR

^b^ Adjusted OR for maternal age, single, parity, number of incomes and maternal education

Overall, infants born to unregistered women with a nationality from outside of the EU were significantly more at risk of perinatal mortality, compared to infants born to registered immigrants (pooled OR(CI95%): 1.5 (1.1–2.1), *p* = 0.02).

We observed a significant excess of prematurity among unregistered mothers in all nationality groups except for Turkish and South American mothers (Table 4). The nationality groups of unregistered mothers with the strongest risks of prematurity were EU15 and EU27 (OR(95%CI) 2.2 (1.94–2.4) and 2.2 (1.9–2.5) respectively *p* < 0.0001), followed by Maghreb (OR(95%CI) 1.7(1.4–2.1), p < 0.0001). When adjusting for sociodemographic characteristic, the Odd Ratios decreased slightly but remained significant, with the exception of eastern European mothers.

Overall, unregistered women from non-EU countries were more likely to give birth prematurely, compared to registered immigrants from these countries (pooled OR(CI95%): 1.4 (1.2–1.6), *p* < 0.0001) (Supplementary data 3) .

## Discussion

To our knowledge, this is the first study which analyses perinatal health outcomes in a unique and exhaustive Belgian population-based database which includes mothers without legal residence in Belgium (unregistered in NPR). Our research has four main findings: 1) Over the study period, almost 2% of births were to mothers who were not registered in the NPR 2) NPR registration status has a crucial role for perinatal health; 3) NPR-unregistered women with a nationality from Belgium, EU15, EU27, and pooled outside-EU27 had higher unadjusted rates of prematurity, LBW, and perinatal mortality, compared to registered mothers; 4) The excess of perinatal mortality for non-European unregistered mothers could partly be explained by their more precarious socioeconomic situations.

Although unregistered women overall appear to have increased risks of adverse perinatal outcomes, it is likely that the causal mechanisms differ starkly between Belgian, European and non-European women. Below, we put forward some hypotheses about possible mechanisms; these should be corroborated with future research studies.

### Belgian women

All Belgian women having an official residence in Belgium are registered in the National Registry, thus only (some) Belgians living abroad and those without stable accommodation (such as the homeless) are absent from the register. The socioeconomic profile of unregistered Belgian women (slightly older age, less having a very low educational level, and more living alone) leads us to hypothesise that this group represents women studying or working abroad. Their higher prevalence of complications during pregnancy, such as hypertension and diabetes, compared with registered Belgian women, the higher risk of congenital anomalies, and of adverse outcomes including premature birth and perinatal mortality (Supplementary Table 2), points towards a particular group of women with high-risk pregnancies choosing to return to their home country to give birth or to have a late termination of pregnancy. This might be the case especially for women living in a country where perinatal healthcare for high-risk pregnancies is less developed or less accessible. Belgians living abroad often conserve their access to Belgian healthcare services either via public or private health insurance. From a socioeconomic and rights perspective, this group of unregistered Belgian women is not deemed to be particularly vulnerable. However, one needs to consider that the extra high-risk births might represent an additional burden on the healthcare system.

Among the unregistered Belgian women there may be few, yet extremely vulnerable, women who live in Belgium but lack stable residency, and who, consequently, are excluded from social and healthcare rights. From a public health perspective, it would be crucial to identify this group and tackle their needs.

### Women from EU15 countries

Our results show that the prevalence of congenital anomalies was four times higher in EU15 NPR-unregistered mothers than in their registered counterparts. We hypothesise that the high perinatal mortality (3.6% vs. 0.51% for their registered counterparts) and elevated prematurity rates (11.7% vs. 5.75%) among unregistered women from EU15 countries partly represent European women temporarily coming to Belgium because of high-risk pregnancies or, more likely, to have a late termination of pregnancy, which would be more difficult to access in their home countries [17]. In Belgium, late terminations of pregnancy are allowed either when the mother’s health is severely put at risk by the pregnancy or when it is known that the baby will be affected by a severe and incurable illness, this latter notion being somewhat subject to interpretation. In practice, late terminations are routinely carried out [18]. In contrast, late terminations in some of the other EU countries are less easily accessible, either because the legislation is more restrictive or because there is a high refusal by doctors to perform these procedures [17, 18].

Women with a nationality from EU15 countries might also have been absent from the NPR because failing to fulfill the registration criteria (stable residency and adequate financial situation).

### Women with a nationality from newer EU member states

Unregistered women from newer EU member states have a socioeconomic profile that differs considerably to that of women from EU15 countries. One out of five women are under the age of 20, 18% have not continued school after primary level, and more than half don’t have any household incomes. The proportion of unregistered women was particularly high among women from Romania (9.1%).

This group of unregistered women likely includes overstaying vulnerable EU citizens such as “Roma” minorities, who are very unlikely to be granted refugee status, and who often struggle to get legal residency permits which would require having stable residency and fulfilling strict financial and administrative criteria. Data shows that there is an increasing work-related immigration to Belgium from newer EU member states (e.g. Romania and Poland), which leads women to work in the informal or semi-formal economy, often doing cleaning or caring jobs, which are physically demanding and have poor working conditions [19]. Our results show that for women of newer EU27 member states the increased rates of prematurity and perinatal mortality are largely explained by their socioeconomic vulnerability.

### Non-European women

Unregistered women with a nationality from outside of the EU are most likely undocumented (without legal residency permit), with only a small part possibly being less vulnerable “medical tourists”. Studies from other countries have shown that undocumented pregnant women tend to be younger, less cohabiting, and have lower incomes [20]. It has also been noted that UMs use contraception and family planning services less and are thus more likely to have unintended pregnancies, which in turn, has been linked to poorer maternal and child health outcomes [10]. The literature indicates, with few exceptions, that undocumented migrants are more at risk of adverse perinatal health outcomes such as maternal morbidity, preterm birth, and low birth weight both compared to non-migrants and to documented migrants [8, 10, 14]. A study carried out in Sweden revealed that undocumented migrant women were even more vulnerable than other migrant groups, including refugees, who are already a particularly disadvantaged group [21]. Likely explanations for this excess risk include 1) perilous migration 2) exposure to poor living and working conditions and psychosocial stress (migration- and poverty-related) [22] and 3) hampered access to adequate healthcare. UMs live in exile and have mostly left their home country due to harsh living conditions. Many have reached their new home country through perilous journeys and been exposed to traumatic events. For pregnant women, this alone can have an important impact on their health [23, 24].

In Belgium, lacking registration in the NPR has widespread social, economic and psychological implications for UMs. The exclusion from legal employment and social welfare means that their lives are often dictated by poverty and instability. Access to good-quality housing is often prohibitive, and employment opportunities are limited to the black market, where working conditions are poor and wages are low. Environmental exposure to risk factors in the living and working environment, together with the psychosocial stress caused by poverty, instability, isolation and by the anxiety and discrimination [12, 25] that results from leading “invisible” lives, can all adversely affect the mental and physical health of mothers and their unborn babies [24, 26–28].

In Belgium, UMs are excluded from the right to be legally employed and thus to be protected from exploitation and guaranteed adequate pay. They cannot receive social welfare or benefit from social services, their access to adequate housing is strongly hampered by financial insecurity, and they can only access healthcare through a complex administrative procedure.

At EU-level, UMs’ access to healthcare varies significantly, and despite the human rights conventions and common EU policy, many countries still provide only limited care [29]. A recent systematic review revealed that UMs were found to be much more likely to have infrequent, late or no antenatal care, compared to legal residents or documented migrants [10].

Legal access restrictions are likely the first and most limiting barrier; yet, beyond this, other reasons cause UMs not to seek help or not to be able to access adequate medical care [9, 30]. At structural level, there are bureaucratic barriers (complex administrative procedures, need for documents), or lack of healthcare adaptation to migrants (lack of translation services or cultural competency). At individual level, there can be fear of being reported to authorities, shame, communication barriers, lack of knowledge concerning the functioning of the healthcare system or unawareness of own rights, and differences in belief systems such as valuing preventive care less [9–11, 30]. Lower use of perinatal services among undocumented migrants has been linked to adverse health outcomes for mothers and babies in studies carried out in the Netherlands, Spain, Switzerland, and Portugal [10]. In Belgium, UMs are excluded from the insurance-based public health coverage. They are theoretically entitled to receiving free healthcare but not without first applying for Urgent Medical Care (AMU) through a complex and sometimes long administrative procedure, involving a social enquiry to verify their “illegal” stay and destitution. The misinformation concerning their rights, and the complexity of the procedure often act as a barrier to accessing (timely) healthcare [31]. A study carried out in Belgium showed that undocumented women who were not covered by the AMU, were more likely to deliver preterm and to have babies with lower birthweights, compared to women covered by social security either via the AMU or the public health insurance [32]. Simplifying the procedure to access AMU or giving direct entitlement to the insurance-based public health coverage would help to increase the timely access to prenatal care.

### Unknown category

Between 2010 and 2016, the nationality of around 3% of unregistered mothers was unknown. Among these births, 4.4% ended in decease. This rate is higher than that of any nationality group and is probably partly due to information bias: parents declaring a perinatal death at the civil registration office might be less likely to be asked socioeconomic questions such as their nationality. In our previous studies, we have also observed under-reporting of SES variables in death certificates [4, 6, 7], and other studies have also pointed out the high health risks observed among unknown/missing categories [33].

### Strengths and limitations

The results of this study are based upon an exhaustive linked nationwide dataset including demographic, socioeconomic, migration, morbidity and mortality information for the entire population including women who are absent from the National Population Registry. It is the first study in Belgium which compares the perinatal health outcomes between registered and non-registered women.

A limitation is that we were not able to distinguish between mothers living in Belgium and those living abroad, which prevents us to differentiate between women living “undocumented” and health tourists. Future studies would certainly gain from being able to differentiate the two. Furthermore, information on healthcare access and perinatal healthcare use might allow for a better understanding of the nature of health inequalities. We were also not able to identify births to the same mother within the seven-year period, given that the unique identifier related to births and not to mothers.

Moreover, given that income and education could be a mediating factor between registration status and perinatal outcomes, adjusting for these variables possibly removes some of the causal effect of unregistered status on the outcomes.

A further limitation is that considerable data was missing for sociodemographic and economic characteristics of unregistered women, which significantly reduced the number of records included in the adjusted models. This underlines the importance of enhancing the collection of routine socioeconomic and health data to yield more accurate estimates of the effects of being unregistered.

## Conclusions

Over the study period, almost 2% of births were to mothers who were not registered in the NPR. This is the first available estimate of births outside of the NPR in Belgium.

Measuring the perinatal health outcomes for the births which are invisible in the national statistics has allowed us to reveal the accrued rates of prematurity, low birthweight and perinatal mortality for NPR-unregistered women in most nationality groups. We have put forward hypotheses concerning the explanatory mechanisms, and these should be corroborated by future research studies. Part of the accrued rates of adverse outcomes (especially among NPR-unregistered Belgian women and those from EU15 countries) could be the result of inadequate or hardly accessible perinatal health services for high-risk pregnancies in other countries, driving women to deliver or terminate a pregnancy in Belgium. Another part (especially concerning NPR-unregistered women from newer EU member states and countries outside of the EU) could be the result of the extremely harsh socioeconomic situations and hampered access to healthcare services that are the consequences of living undocumented in Belgium.

We hope that by measuring the invisible we contribute to shedding light on the most marginalised and vulnerable groups of women, who are most in need of socioeconomic integration and access to services, and thus support the fight against the perpetuation of inequalities from the mere start of life.

## Supplementary Information

**Additional file 1: S1 Table.** List of countries included in each nationality category.

**Additional file 2: S2 Table.** Distribution of all singleton births according to maternal sociodemographic characteristics among NPR registered mother.

**Additional file 3: S3 Table.** Low birth weight (crude rates among No maternal NPR) and Odds Ratios according to NPR registration status (reference is maternal NPR), stratified by maternal nationality.

## Data Availability

The data that support the findings of this study are available from Statistics Belgium (DGSIE) but restrictions apply to the availability of these data, which were used under license for the current study, and so are not publicly available. Data are however available from the authors upon reasonable request (judith.racape@ulb.be) and with permission of Statistics Belgium (DGSIE).
