# Supplementary material for: Measuring the invisible: perinatal health outcomes of unregistered women giving birth in Belgium, a population-based study
Source: BMC Pregnancy Childbirth. 2021 Oct 29;21:733. doi: 10.1186/s12884-021-04183-9 (PMC8555314; doi:10.1186/s12884-021-04183-9)
Supplement: Supplementary file 1 — Additional file 1: S1 Table. List of countries included in each nationality category. [file 12884_2021_4183_MOESM1_ESM.docx]

**S1** Table : List of countries included in each nationality category

| ***EU-15*** | ***EU27***  ***(without EU15)*** | ***East Europe and Russia*** | ***Turquie*** | ***Maghreb*** | ***sub Saharan Africa*** | ***South America*** | ***Middle east and North/West Asia*** |
| --- | --- | --- | --- | --- | --- | --- | --- |
| Austria | Bulgaria | Serbia | Turkey | Algeria | Angola | Argentina | Afghanistan |
| Denmark | Croatie | Albania |  | Egypt | Benin | Bahamas | Armenia |
| Finland | Cyprus | Belarus |  | Libya | Botswana | Barbados | Azerbadjan |
| France | Czech Republic | Bosnia-Herzegovina |  | Mauritania | Burkina Faso | Belize | Bahrain |
| Germany | Estonia | Kosovo |  | Morocco | Burundi | Bolivia | Georgia |
| Greece | Hungary | Macedonia |  | Tunisia | Cabo Verde | Brazil | Iran |
| Ireland | Latvia | Moldova |  |  | Cameroon | Chile | Iraq |
| Italy | Lithuania | Montenegro |  |  | Central African Republic | Colombia | Israel |
| Luxembourg | Malta | Republic of Moldova |  |  | Chad | Costa Rica | Jordan |
| Netherlands | Poland | Russian Federation |  |  | Comoros | Cuba | Kazakhstan |
| Portugal | Romania | Ukraine |  |  | Democratic Rep. of Congo | Dominica | Kuwait |
| Spain | Slovakia |  |  |  | Djibouti | Dominican Republic | Kyrgistan |
| Sweden | Slovenia |  |  |  | Equatorial Guinea | Ecuador | Lebanon |
| United Kingdom |  |  |  |  | Eritrea | El Salvador | Oman |
|  |  |  |  |  | Ethiopia | Guatemala | Pakistan |
|  |  |  |  |  | Gabon | Guyana | Palestine |
|  |  |  |  |  | Gambia | Haiti | Saudi Arabia |
|  |  |  |  |  | Ghana | Honduras | Syria |
|  |  |  |  |  | Guinea | Jamaica | Tajikistan |
|  |  |  |  |  | Guinea-Bissau | Mexico | Turkmenistan |
|  |  |  |  |  | Ivory coast | Nicaragua | United Arab Emirates |
|  |  |  |  |  | Kenya | Panama | Uzbekistan |
|  |  |  |  |  | Lesotho | Paraguay | Yemen |
|  |  |  |  |  | Liberia | Peru |  |
|  |  |  |  |  | Madagascar | Puerto Rico |  |
|  |  |  |  |  | Malawi | Saint Lucia |  |
|  |  |  |  |  | Mali | Suriname |  |
|  |  |  |  |  | Mauritius | Trinidad and Tobago |  |
|  |  |  |  |  | Mozambique | Uruguay |  |
|  |  |  |  |  | Namibia | Venezuela |  |
|  |  |  |  |  | Ngwane |  |  |
|  |  |  |  |  | Niger |  |  |
|  |  |  |  |  | Nigeria |  |  |
|  |  |  |  |  | Rwanda |  |  |
|  |  |  |  |  | Sao Tome |  |  |
|  |  |  |  |  | Senegal |  |  |
|  |  |  |  |  | Seychelles |  |  |
|  |  |  |  |  | Sierra Leone |  |  |
|  |  |  |  |  | Somalia |  |  |
|  |  |  |  |  | South Africa |  |  |
|  |  |  |  |  | Sudan |  |  |
|  |  |  |  |  | Tanzania |  |  |
|  |  |  |  |  | Togo |  |  |
|  |  |  |  |  | Uganda |  |  |
|  |  |  |  |  | Zambia |  |  |
|  |  |  |  |  | Zimbabwe |  |  |
