# Supplementary material for: Measuring the invisible: perinatal health outcomes of unregistered women giving birth in Belgium, a population-based study
Source: BMC Pregnancy Childbirth. 2021 Oct 29;21:733. doi: 10.1186/s12884-021-04183-9 (PMC8555314; doi:10.1186/s12884-021-04183-9)
Supplement: Supplementary file 2 — Additional file 2: S2 Table. Distribution of all singleton births according to maternal sociodemographic characteristics among NPR registered mother. [file 12884_2021_4183_MOESM2_ESM.docx]

**S2 Table****: Distribution of all singleton births according to maternal sociodemographic characteristics among NPR registered mother**

| **N=854 689** | **Belgium**  **(n=663 539)** | **EU15**  **(n=54 895)** | **EU27 without EU15**  **(n=27 724)** | **East Europe**  **(n=13 754)** | **Turkey**  **(n=8 212)** | **Maghreb (n=33 570)** | **Sub-Saharan Africa**  **(n=24 932)** | **South America**  **(n= 4 200)** | **Middle east**  **(n= 10 546)** | **Other**  **(n= 10 580)** | **Unknown**  **(n= 2 737)** |
| --- | --- | --- | --- | --- | --- | --- | --- | --- | --- | --- | --- |
| **% of births** | 77.6 | 6.4 | 3.2 | 1.6 | 0.96 | 3.9 | 2.9 | 0.49 | 1.2 | 1.2 | 0.32 |
| **Parity** | 660 037 | 53 690 | 27 534 | 13 662 | 8 154 | 33 346 | 24 709 | 4 163 | 10 450 | 10 494 | 1 459 |
| Nulliparity (%) | 44.4 | 45;0 | 48.0 | 37.7 | 34.65 | 38.1 | 33.5 | 45.6 | 36.8 | 51.2 | 33.7 |
| ≥ 3 children (%) | 7.95 | 8.4 | 5.85 | 16.7 | 8.9 | 11.9 | 15.6 | 6.9 | 14.1 | 4.3 | 22.2 |
| **Living alone** | 658 234 | 52 419 | 27 208 | 13 430 | 8 153 | 33 296 | 24 231 | 4 113 | 10 278 | 10 430 | 1 266 |
| Yes (%) | 11.8 | 10.6 | 12.7 | 17.0 | 5.4 | 5.8 | 37.6 | 13.8 | 12.4 | 6.7 | 34.2 |
| **Maternal age** | 663 539 | 54 895 | 27 724 | 13 754 | 8 212 | 33 570 | 24 932 | 4 200 | 10 546 | 10 580 | 2 055 |
| < 20 (%) | 2.6 | 1.7 | 4.9 | 9.5 | 4.0 | 1.6 | 3.65 | 3.0 | 4.1 | 0.67 | 9.05 |
| ≥ 40 (%) | 1.6 | 3.5 | 1.6 | 1.4 | 1.4 | 3.0 | 2.1 | 3.4 | 1.6 | 2.6 | 2.8 |
| **Maternal education** | 663 539 | 54 895 | 27 724 | 13 754 | 8 212 | 33 570 | 24 932 | 4 200 | 10 546 | 10 580 | 2 055 |
| < primary (%) | 2.3 | 3.2 | 9.6 | 20.7 | 21.3 | 22.5 | 19.9 | 7.3 | 21.3 | 9.2 | 13.8 |
| secondary (%) | 43.5 | 38.4 | 46.5 | 39.05 | 50.9 | 47.4 | 44.5 | 49.4 | 35.2 | 33.3 | 19.6 |
| tertiary (%) | 44.3 | 39.6 | 27.1 | 15.1 | 6.3 | 10.8 | 13.1 | 27.1 | 15.9 | 35.85 | 2.7 |
| Other (%) | 9.9 | 18.8 | 16.8 | 25.2 | 21.4 | 19.3 | 22.5 | 16.2 | 27.7 | 21.7 | 63.9 |
| **Number of incomes** | 624 399 | 46 184 | 24 620 | 11 477 | 7 298 | 29 696 | 21 177 | 3 661 | 8 539 | 9 187 | 1 020 |
| 0 incomes (%) | 10.5 | 10.8 | 13.1 | 43.0 | 24.4 | 25.9 | 46.7 | 15.8 | 42.0 | 11.95 | 66.7 |
| 1 income (%) | 20.1 | 30.2 | 38.9 | 42.3 | 64.1 | 63.6 | 40.8 | 45.6 | 47.2 | 55.0 | 28.3 |
| 2 incomes (%) | 69.3 | 59.0 | 48.0 | 14.7 | 11.5 | 10.5 | 12.6 | 38.6 | 10.8 | 33.1 | 5.0 |
| **Congenital anomalies** | 656 898 | 50 009 | 27 139 | 13 564 | 8 111 | 33 181 | 24 575 | 4 089 | 10 374 | 10 282 | 1 329 |
| Yes (%) | 0.69 | 0.65 | 0.64 | 0.75 | 1.0 | 0.88 | 0.72 | 0.68 | 0.74 | 0.62 | 1.05 |
| **Hypertension** | 654 683 | 49 721 | 27 017 | 13 466 | 8 077 | 33 070 | 24 467 | 4 076 | 10 311 | 10 250 | 1 321 |
| Yes (%) | 4.8 | 3.6 | 3.4 | 2.6 | 2.7 | 2.2 | 6.9 | 3.9 | 2.2 | 2.5 | 3.8 |
| **Diabetes** | 652 441 | 49 529 | 26 845 | 13 357 | 8 029 | 32 848 | 24 261 | 4 062 | 10 238 | 10 218 | 1 329 |
| Yes (%) | 4.85 | 5.4 | 5.1 | 4.3 | 6.0 | 10.7 | 7.8 | 6.8 | 6.7 | 7.0 | 8.7 |
| **Delivery Mode** | 656 112 | 49 916 | 27 125 | 13 557 | 8 107 | 33 165 | 24 559 | 4 088 | 10 365 | 10 267 | 1 327 |
| Vaginal (%) | 80.6 | 79.3 | 80.9 | 84.5 | 81.9 | 81.6 | 69.1 | 70.65 | 79.4 | 74.6 | 78.3 |
| Caesarean (%) | 10.0 | 10.4 | 9.5 | 7.7 | 8.9 | 8.3 | 13.5 | 15.4 | 10.9 | 11.8 | 10.85 |
| Emergency caesarean (%) | 9.4 | 10.3 | 9.5 | 7.8 | 9.2 | 10.1 | 17.4 | 14.0 | 9.6 | 13.7 | 10.85 |
| **Perinatal mortality** | 663 539 | 54 895 | 27 724 | 13 754 | 8 212 | 33 570 | 24 932 | 4 200 | 10 546 | 10 580 | 2 737 |
| Yes (%) | 0.51 | 0.51 | 0.53 | 0.52 | 0.74 | 0.76 | 0.91 | 0.69 | 0.77 | 0.53 | 25.4 |
| **Low birth weight** | 658 464 | 50 096 | 27 228 | 13 609 | 8 143 | 33 314 | 24 704 | 4 106 | 10 426 | 10 307 | 1 999 |
| Yes (%) | 5.5 | 5.0 | 4.5 | 4.3 | 4.9 | 3.7 | 6.6 | 5.0 | 5.35 | 5.3 | 37.2 |
| **Prematurity** | 658 610 | 50 113 | 27 235 | 13 617 | 8 149 | 33 337 | 24 730 | 4 107 | 10 430 | 10 312 | 2 011 |
| Yes (%) | 6.8 | 5.75 | 5.9 | 5.5 | 5.8 | 4.45 | 7.1 | 6.7 | 5.7 | 6.0 | 37.1 |
