# Supplementary material for: Measuring the invisible: perinatal health outcomes of unregistered women giving birth in Belgium, a population-based study
Source: BMC Pregnancy Childbirth. 2021 Oct 29;21:733. doi: 10.1186/s12884-021-04183-9 (PMC8555314; doi:10.1186/s12884-021-04183-9)
Supplement: Supplementary file 3 — Additional file 3: S3 Table. Low birth weight (crude rates among No maternal NPR) and Odds Ratios according to NPR registration status (reference is maternal NPR), stratified by maternal nationality. [file 12884_2021_4183_MOESM3_ESM.docx]

**S3 Table : Low birth weight (crude rates among No maternal NPR) and Odds Ratios according to NPR registration status (reference is maternal NPR), stratified by maternal nationality**

*1. Crude OR*

*2. Adjusted OR for maternal age, single, parity, number of incomes and maternal education*

| **Maternal Nationality** | **Per 100 births** | **OR^1^** | **CI (95%)** | ***p-value*** | **aOR^2^** | **CI (95%)** | ***p-value*** |
| --- | --- | --- | --- | --- | --- | --- | --- |
| **Belgium** |  | cases=36 717 / n= 661 551 | | | cases=33 776 / n= 626 281 | | |
|  | 9.6 | **1.8** | **1.6-2.03** | ***<0.0001*** | **1.5** | **1.35-1.8** | ***<0.0001*** |
| **EU15** |  | cases=2 952 / n= 54 313 | | | cases=2 526 / n= 49 787 | | |
|  | 10.4 | **2.2** | **2.0-2.4** | ***<0.0001*** | **1.6** | **1.4-1.9** | ***<0.0001*** |
| **EU27 (without EU15)** |  | cases=1 430 / n= 29 544 | | | cases=1 216 / n= 25 396 | | |
|  | 8.8 | **2.0** | **1.75-2.4** | ***<0.0001*** | **1.45** | **1.2-1.8** | ***<0.0001*** |
| **Eastern Europe** |  | cases=685 / n= 15 034 | | | cases=532 / n= 12 465 | | |
|  | 7.0 | **1.7** | **1.35-2.1** | ***<0.0001*** | **1.4** | **1.1-1.9** | ***0.01*** |
| **Turkey** |  | cases=409 / n= 8 369 | | | cases=368/ n= 7 458 | | |
|  | 4.0 | 0.8 | 0.4-1.6 | *0.52* | 0.9 | 0.4-1.9 | *0.72* |
| **Maghreb** |  | cases=1 295 / n= 34 624 | | | cases=1 112 / n= 30 597 | | |
|  | 5.65 | **1.6** | **1.2-2.0** | ***<0.0001*** | 1.3 | 0.95-1.8 | *0.11* |
| **Sub-Saharan Africa** |  | cases=1 787 / n= 26 142 | | | cases=1 451 / n= 22 255 | | |
|  | 10.2 | **1.6** | **1.35-1.9** | ***<0.0001*** | **1.4** | **1.1-1.7** | ***0.002*** |
| **South America** |  | cases=249 / n=4 920 | | | cases=286 / n=4 283 | | |
|  | 5.5 | 1.1 | 0.8-1.6 | *0.51* | 0.98 | 0.7-1.4 | *0.91* |
| **Middle East and North/West Asia** |  | cases=601 / n=11 004 | | | cases=463 / n=8 952 | | |
|  | **7.4** | **1.4** | **1.0-1.95** | ***0.03*** | **1.5** | **1.0-2.2** | ***0.04*** |
| **Other** |  | cases=597 / n=10 929 | | | cases=519 / n=9 640 | | |
|  | 7.7 | **1.5** | **1.1-2.0** | ***0.01*** | **1.7** | **1.2-2.4** | ***0.004*** |
| **Outside EU (Pool)** |  | cases=5 103 / n=102 039 | | | cases=4 207 / n=87 720 | | |
|  | 7.3 | **1.5** | **1.4-1.7** | ***<0.0001*** | **1.3** | **1.15-1.5** | ***<0.0001*** |
